# Development and Validation of a Smartphone-based Contrast Sensitivity Test

**DOI:** 10.1167/tvst.8.5.13

**Published:** 2019-09-13

**Authors:** Esmael Habtamu, Andrew Bastawrous, Nigel M. Bolster, Zerihun Tadesse, E. Kelly Callahan, Bizuayehu Gashaw, David Macleod, Matthew J. Burton

**Affiliations:** 1 International Centre for Eye Health, London School of Hygiene & Tropical Medicine, London, UK; 2 Peek Vision Limited, London, UK; 3 The Carter Center, Addis Ababa, Ethiopia; 4 The Carter Center, Atlanta, GA, USA; 5 Amhara Regional Health Bureau, Bahirdar, Ethiopia

**Keywords:** contrast sensitivity, smartphone, trachoma

## Abstract

**Purpose:**

Contrast sensitivity (CS) testing is an important measure of visual function reflecting variations in everyday visual experience in different conditions and helps to identify more subtle vision loss. However, it is only infrequently used. To make this more accessible, we have developed and validated a smartphone-based CS test.

**Methods:**

A new tumbling-E smartphone-based CS test was developed, Peek Contrast Sensitivity (PeekCS). This was field tested and refined through several iterations. Reference standard was a tumbling-E Pelli-Robson CS test (PRCS). The validation study was conducted in community clinics in Ethiopia. Test-retest variability was measured for both PRCS and PeekCS. PRCS and PeekCS were then compared. Correlation coefficients and 95% confidence intervals (CIs) were calculated; 95% limits of agreement were calculated and displayed on Bland-Altman plots.

**Results:**

PeekCS showed strong repeatability (correlation coefficient: 0.93; 95% CI: 0.91–0.95), which was comparable with PRCS (correlation coefficient: 0.96; 95% CI: 0.95–0.97). The 95% limit of agreement for test-retest variability of PRCS and PeekCS were −0.20 to 0.21 and −0.31 to 0.29, respectively. PRCS and PeekCS were highly correlated: 0.94 (95% CI: 0.93–0.95); 95% limits of agreement −0.27 to 0.29; and mean difference 0.010 (95% CI: −0.001 to 0.022). PeekCS had a faster testing time (44.6 seconds) than PRCS (48.6 seconds): mean difference −3.98 (95% CI: −5.38 to −2.58); *P* < 0.001.

**Conclusions:**

The smartphone-based PeekCS is a repeatable and rapid test, providing results that are highly comparable with the commonly used PRCS test.

**Translational Relevance:**

PeekCS provides an accessible and easy to perform alternative for CS testing, particularly in the community setting.

## Introduction

Visual acuity (VA) is the most frequently performed measure of visual function. In a VA test, optotypes of decreasing size with a fixed high contrast level are presented. However, high contrast does not always reflect performance in real world situations. Contrast sensitivity (CS), an important measure of visual function, is the ability to detect a difference between the luminance of an object and its background.^1,2^ The varying levels of contrast presented in a CS test more accurately represent variations common to everyday visual experience.^3,4^

Poor CS degrades quality of vision, by reducing the ability to distinguish between objects without distinct outlines, affecting day to day activities even in people with normal VA.^1,3,5–8^ CS is a useful measure of visual function in evaluating patients with cataract, glaucoma, diabetic retinopathy, and macular degenerations; the leading causes of blindness worldwide.^9–12^ It is also an important measure of visual function for occupations requiring particularly good eye sight. Poor CS can significantly limit activity and reduce quality of life.^2,12^ In a recent study from Ethiopia, poor CS was strongly associated with reduced quality of life scores in patients with trachomatous trichiasis.^13^

CS tests usually involve letters of a fixed size, which gradually become lighter through the test, until they are almost identical to background and impossible to detect.^1,14^ There are several chart and computer-based CS tests. Perhaps the most widely used is the Pelli-Robson Contrast Sensitivity (PRCS) test.^14^ This provides a reliable and repeatable measure of low spatial frequency CS, tested at 1 meter. It has been used in multiple studies as the reference standard for evaluating other CS tests.^15,16^ The PRCS chart can be produced in a “tumbling E” format for use in a context with a low literacy level.

CS is infrequently measured in routine clinical practice, for several reasons: lack of familiarity, time constraints, interpretation difficulty or unavailability. PRCS is large and needs careful handling; therefore, it is less easy to use in outreach clinics. Other tests of CS, such as the Spaeth/Richman Contrast Sensitivity (SPARCS) test, require a computer with internet access, making them impractical for outreach or a low resource setting.^15,16^

Increased availability of smartphones is transforming vision measurement and access to eye care services in hard to reach low-income settings.^17^ Peek Vision is developing smartphone-based tools to address these needs (https://www.peekvision.org). The Peek Acuity app, which measures distance VA, has been found to be repeatable and reliable.^17^ Furthermore, the app's inclusion in a community vision screening program in rural Kenya demonstrated the robustness of smartphone-based tests in such settings as well as their ability to remain charged throughout an entire day's testing.

Various mobile electronic device based CS tests have been studied and developed.^18–25^ However, all of the tests designed for mobile devices were written for iOS and validated using Apple (Cupertino, CA) products.^18–24^ As of June 2019, the most basic models of the latest Apple smartphone, tablet, and MP4 player models (the iPhone 8, iPad, and iPod Touch) were 600, 400, and 200 USD, respectively (www.apple.com). Such costs are prohibitive for at-scale use in low-resource settings. As such these tend to represent a small fraction of the mobile device market in low-income countries, with Apple accounting for less than 4% of the mobile phone market in Ethiopia, for example (http://gs.statcounter.com/vendor-market-share/mobile/ethiopia). Smartphones running the Android operating system are widespread and constitute the vast majority of the market in low-income countries. They are also comparatively inexpensive with devices being available for under 26 USD (www.walmart.com/ip/Tracfone-Alcatel-Raven-Prepaid-Smartphone/613852626). A CS test designed for Android devices is therefore necessary if such a test is to be sustainably introduced into clinical practice at scale in low-income countries. Furthermore, the aforementioned studies were each conducted in high resource settings in relatively small numbers of participants, with all but one having *n* ≤ 40. A smartphone-based CS test designed for and validated in a low-income country is therefore desirable.

In addition, most of these studies used either Quick CSF (a computerized monitor-based test from a Bayesian adaptive procedure) or swept-frequencies or gratings, which probably are not as familiar as optotype-based tests either to a nonliterate patient or primary health care professional in a resource-limited setting, owing to their similarity to established VA tests. Moreover, there is limited evidence on the validity and applicability of the various commercially available mobile device-based CS test applications in nonliterate communities. Furthermore, subtle differences in testing methods such as viewing distance and lighting conditions, or size and screen brightness of different devices, or the variability of settings including the awareness and skill of the persons being tested and doing the test would provide varying results and would affect reliability of CS tests, indicting that more CS tests and applications need to be developed using various methods and in different settings. There is a need for a relatively simple and easy to use CS testing method, particularly by health cadres with limited training in community-based efforts, to streamline comprehensive eye care service delivery.

In this study, we developed a new smartphone-based CS test and validated relative to the PRCS test for use at any level of the health care system, particularly in low-resource settings, and validated this in a study population with a low level of literacy. We refer to this new test as Peek Contrast Sensitivity (PeekCS). The rationale was to produce a smartphone CS test with sufficient accuracy to make CS testing much more widely available, easier, and potentially faster to perform across all settings.

## Methods

This study was conducted in Ethiopia. It was approved by Ethiopian National Health Research Ethics Review Committee, London School of Hygiene & Tropical Medicine Ethics Committee, and Emory University Institutional Review Board. It was conducted in accordance with the Declaration of Helsinki. Written informed consent was obtained from all participants in Amharic before enrolment. Illiterate participants were read the information sheet and consent form; their consent was recorded by thumb print in the presence of a witness. This study was nested within a previously reported randomized placebo controlled trial of oral doxycycline for the prevention of postoperative trichiasis.^26^

### Study Participants

Participants enrolled into the clinical trial were identified through community-based screening, and by organizing community-based surgical outreaches in health facilities in West Gojjam Zone, Amhara Region. Detailed methodology for identification, recruitment, and follow-up of participants has been previously described.^26^ In summary, adults >18 years with trachomatous trichiasis were identified, received surgical treatment, and immediately randomized to receive either oral doxycycline 100 mg per day for 28 days or placebo capsules. Follow-up was conducted at 10 days, and at 1, 6, and 12 months to examine for postoperative trichiasis, corneal opacity, and vision change. The CS test development and validation were conducted during the final 12-month follow-up. The data from the final validation were collected between April 4, and May 7, 2017.

### PeekCS Test Development

The development of the smartphone-based PeekCS test went through multiple stepwise iterations. The development process is described in the Supplementary Material. Within each development cycle, we tested performance in a new group of study participants. As the reference test, we used two separate 1-meter “Tumbling – E” PRCS charts (Precision Vision, Woodstock, IL) ordered and made specifically for use in this study population with low literacy level, following the standard instruction and described in the Supplementary Material.

### Final PeekCS Test

The final PeekCS test version was performed using a Sony Xperia Z3 (Android 4.4). The settings for each CS are shown in Table 1. The smartphone was mounted on a tripod with a “Twist Grip” clamp. Eyes were tested separately at 1 meter. Screen brightness was set to 100%. One letter “E” was displayed at a time in one of four random orientations. The tester swiped the screen in the direction the participant indicated, to record the response. The test logic and method are described in detail in Online Supplementary Material and Figure S2. At the end of the test, the application displays the log CS result.

**Table 1:** The RGB Values Used in the PeekCS Test^a^

Score Stage	Optotype Gray RGB Value	Background Gray RGB Value	Actual Peek CS Log_10_ (CS)	Standard Error	Pelli-Robson Log_10_ (CS)	Contrast
1	0	255	0.006	0.000007	0.00	Highest
2	164	255	0.20	0.00040	0.15	E
3	197	254	0.35	0.000625	0.30	
4	216	255	0.50	0.00026	0.45	
5	229	255	0.65	0.001225	0.60	
6	237	255	0.78	0.00306	0.75	
7	242	254	0.96	0.00208	0.90	
8	246	255	1.06	0.03935	1.05	
9	248	255	1.16	0.0017	1.20	
10	250	254	1.40	0.01185	1.35	
11	250	253	1.53	0.0184	1.50	
12	252	255	1.67	0.00475	1.65	
13	246	248	1.71	0.00875	1.80	
14	252	254	1.94	0.0034	1.95	
15	254	255	2.00	0.0076	2.10	**E**
16	N/A	N/A	N/A	N/A	2.25	Lowest

N/A, not applicable; RGB, red, green, blue.

a Designed for a Sony Xperia Z3 for each Score Stage and the corresponding actual CS measured by a photometer in a darkened laboratory setting. Owing to the finite combinations of optotype and background grays, it is not possible to have an exact alignment between the contrast sensitivities tested with PeekCS and the Pelli-Robson charts. However, the difference between the two tests is never more than two-thirds of the difference between score stages (i.e., 0.10 log units).

### Validation Procedures

All CS tests were conducted prior to any ocular examination. Four CS tests were performed for each eye separately: two PRCS (different charts) and two PeekCS. Each test was completed for right eye and then left eye before doing the next test. Test order was chosen at random using a computer-generated random table. The four possible testing orders were: (1) PeekCS1, PRCS1, PeekCS2, PRCS2; (2) PRCS1, PeekCS1, PeekCS2, PRCS2; (3) PeekCS1, PRCS1, PRCS2, PeekCS2; (4) PRCS1, PeekCS1, PRCS2, PeekCS2. A single health officer conducted both tests in the same room with adequate natural light. Ambient light was measured using an ISO-TECH ILM 1332A Lux meter for the adjacent places where the Pelli-Robson charts and smartphone were positioned for testing. The PeekCS app also displays an alert if the phone's integrated light sensor detected an ambient light level exceeding 900 lux. Participants were given sufficient time to identify the direction of each letter, particularly when near threshold. VA was measured. Eyes were examined with 2.5× loupes and torch for signs related to trachoma including trichiasis and corneal opacification. The detailed examination methodology has been previously described.^26^

### Statistical Analysis

Data were double-entered into Access (Microsoft), cleaned, and transferred to Stata 14.2 (StataCorp) for analysis. Demographic and clinical data were summarized using means and proportions. Data from both right and left eyes are used in this analysis.

Test-retest variability was analyzed for PRCS1 versus PRCS2 and PeekCS1 versus PeekCS2. PRCS was compared with PeekCS by combining the first and second test pairs of each. Correlation coefficients and 95% CIs were generated to determine linear relationship between tests. Scatter plots were used to plot the distribution comparisons between tests. Bland-Altman plots were generated from the individual's mean CS score measured by the two tests being compared and the difference in CS between the two test results. The 95% CI limits of agreement were calculated as the mean difference between tests ± 1.96 multiplied by the standard deviation of the mean differences.^27^ Mean differences in CS, test times, and room brightness were estimated using mixed effect linear regression models. A mixed effect model was used to account for the fact that each participant contributed CS tests from both eyes, and when comparing PeekCS and PRCS, each eye contributed two CS tests, so the model included random intercepts for both patient and eye, with eye nested within patient.

## Results

CS was measured using two PRCS charts and the final version of PeekCS for 147 individuals (294 eyes). Demographic and clinical characteristics are described in Table 2. Participants had a mean age of 50.3 years (range, 18–82 years) and were predominantly female (110/147 [74.8%]) and illiterate (128/147 [87.1%]). All participants had received corrective upper eyelid surgery for trachomatous trichiasis in at least one eye 12 months prior to this data collection: 50 (34%) right eyelid surgery only, 73 (49.7%) left eyelid surgery only, and 24 (16.3%) both eyelids. Overall, 171/294 (58.2%) of the eyes tested for CS had previously undergone trichiasis surgery. Among the 294 eyes, 67 (22.8%) had trichiasis at 12 months, of which the majority (60/67 [89.5%]) were minor trichiasis (one to five lashes touching the eye). Some degree of corneal opacity was reported in two thirds of eyes (Table 2). In 69 (32.5%) eyes, there was central corneal opacity and 60 (20.4%) eyes had off center opacities, encroaching within the central 4 mm. Eight (2.7%) eyes had easily visible mature cataract. VA was impaired in 101 (34.4%) eyes (Table 2).

**Table 2:** Demographic and Clinical Characteristic of Participants

Characteristics	No. (%)
Demographic, *n* = 147	
Age, mean ± SD, y	50.3 ± 13.7
Female	110 (74.8)
Illiterate	128 (87.1)
Eye, *n* = 294	
VA	
Normal (≥6/18)	193 (65.6)
Moderate visual impairment (<6/18 to ≥6/60)	95 (32.3)
Severe visual impairment (<6/60 to ≥3/60)	2 (0.7)
Blind (<3/60)	4 (1.4)
Previous trichiasis surgery	
No	123 (41.8)
Yes	171 (58.2)
Trichiasis (number of lashes currently touching eye)	
No trichiasis	227 (77.2)
None (epilating)	3 (1.0)
1–5	57 (19.4)
6–9	5 (1.7)
10–19	2 (0.7)
Corneal opacity	
None (CC0)	99 (33.7)
Peripheral (CC1)^a^	66 (22.5)
Off center, faint (CC2a)^b^	57 (19.4)
Off center, dense (CC2b)^b^	3 (1.0)
Central, faint (CC2c)^c^	60 (20.4)
Central, dense (CC2d)^c^	9 (3.1)
Total central, dense (CC3)	0 (0.0)
Phthisis (CC4)	0 (0.0)

Faint indicates pupil margin is visible through the opacity; dense indicates the pupil margin is not visible through the opacity.

a Peripheral is outside the central 4 mm.

b Off center is within the central 4 mm but does not involve the central 1 mm.

c Central involves the central 1 mm of the cornea.

**Figure 1:**
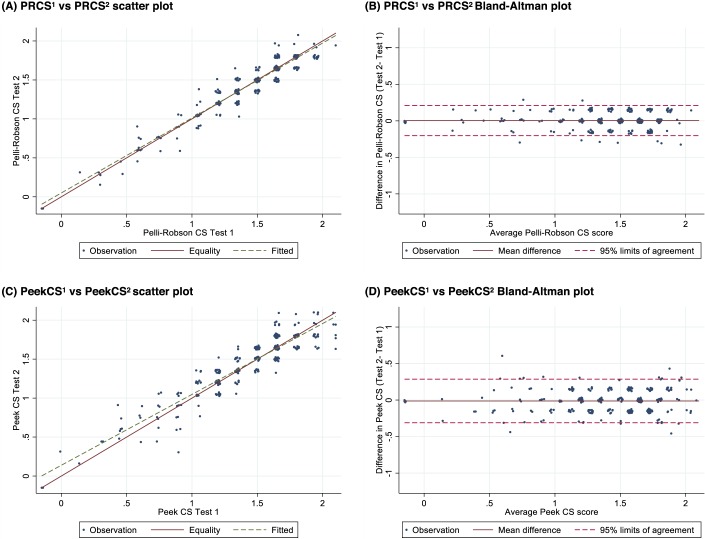
Scatter and Bland-Altman plots for test-retest: (A, B) Pelli-Robson CS (PRCS1 versus PRCS2) and (C, D) Peek Contrast Sensitivity (PeekCS1 versus PeekCS2).

The comparison between the four tests repeatability and correlation results are presented in Table 3. The PRCS test had very strong test-retest characteristics with a correlation of 0.96 (95% CI: 0.95–0.97) and no evidence of systematic difference in mean (“bias”) between the two tests (mean difference: 0.004; 95% CI: −0.008 to 0.016). The scatter plot (Fig. 1a) shows that the fitted line is very close to the line of equality. The 95% limits of agreement in the Bland-Altman analysis (Fig. 1b) were between −0.20 and 0.21, indicating that in 95% of repeated tests the difference would be less than two PRCS steps. In this analysis, 59.5% of paired observations were identical, and a further 37.4% scored only one step difference.

**Table 3:** Repeatability and Correlation of CS Tests^a^

Tests	Mean (95% CI)	Mean Difference (95% CI)	95% Limit of Agreement
PRCS1 vs. PRCS2			
PRCS1	1.407 (1.349 to 1.465)	0.004 (−0.008 to 0.016)	−0.20 to 0.21
PRCS2	1.403 (1.345 to 1.461)		
PeekCS1 vs. PeekCS2			
PeekCS1	1.389 (1.328 to 1.450)	−0.012 (−0.030 to 0.005)	−0.31 to 0.29
PeekCS2	1.401 (1.340 to 1.462)		
PRCS vs. PeekCS^b^			
PRCS	1.405 (1.346 to 1.464)	0.010 (−0.001 to 0.022)	−0.27 to 0.29
PeekCS	1.395 (1.336 to 1.454)		

a All analyses are adjusted for bilateral eye data using mixed effects models.

b Combined analysis of the first and second PRCS and PeekCS pairs, adjusted using mixed effects models.

**Table 3:** Extended

Tests	Pearson Correlation Coefficient (95% CI)	Intraclass Correlation Coefficient (95% CI)
PRCS1 vs. PRCS2		
PRCS1	0.96 (0.95 to 0.97)	0.96 (0.95 to 0.97)
PRCS2		
PeekCS1 vs. PeekCS2		
PeekCS1	0.93 (0.91 to 0.95)	0.93 (0.91 to 0.95)
PeekCS2		
PRCS vs. PeekCS^b^		
PRCS	0.94 (0.93 to 0.95)	0.94 (0.92 to 0.95)
PeekCS		

PeekCS showed strong repeatability with a correlation of 0.93 (95% CI: 0.91–0.95). There was no evidence of bias between the two PeekCS tests (mean difference: −0.012; 95% CI: −0.030 to 0.005). The scatter plot and Bland-Altman for PeekCS repeatability are shown in Figures 1c and 1d. The 95% limit of agreement between PeekCS1 and PeekCS2 were slightly wider than the PRCS tests at −0.31 to 0.29. This is roughly equivalent to two PRCS steps, with 38.4% of paired observations being identical, and a further 50.7% scoring only one step difference between the two PeekCS tests.

**Figure 2:**
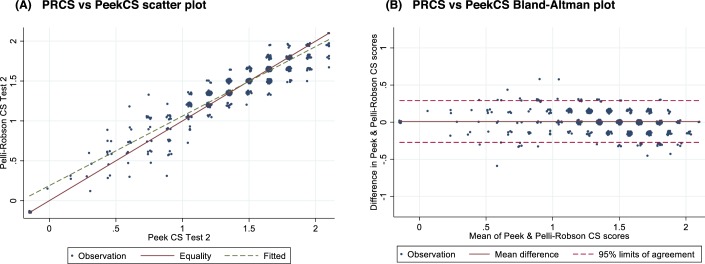
(A) Scatter plot and (B) Bland-Altman plot for PRCS and PeekCS.

Combining the two sets of PRCS versus PeekCS comparisons (PRCS1 versus PeekCS1 and PRCS2 versus PeekCS2) found a high degree of correlation 0.94 (95% CI: 0.93–0.95) (Table 3). The Bland-Altman 95% limits of agreement were −0.27 and 0.29, equivalent to two PRCS steps; with 45.6% of paired observations being identical, and a further 44.7% scoring only one step difference between the two tests (Fig. 2). The estimated mean difference between PRCS and PeekCS was 0.010 (95% CI: −0.001 to 0.022), with the slightly higher mean score in PRCS. The upper bound of this CI represents only approximately one-seventh of a PRCS step.

Test time for PeekCS was faster than PRCS: mean PRCS test time was 48.6 seconds and PeekCS was 44.6 seconds (mean difference: −3.98; 95% CI: −5.38 to −2.58; *P* < 0.001). The second round of testing was significantly faster for both PRCS (PRCS1 versus PRCS2 mean difference: −8.29; 95% CI: −9.63 to −6.94; *P* = <0.0001) and PeekCS (PeekCS1 versus PeekCS2 mean difference: −7.41; 95% CI: −9.34 to −5.49; *P* = <0.0001).

There was no difference in mean room illuminance between the first and the second PeekCS tests (mean 303.7 lux versus 304.2 lux; mean difference: 0.46; 95% CI: −4.86 to 5.78; *P* = 0.865) and the first and second PRCS tests (mean 294.2 lux versus 297.6 lux; mean difference: 3.45; 95% CI: −2.18 to 9.07; *P* = 0.230). The average room brightness was slightly higher for PeekCS compared with PRCS (mean difference: 8.06 lux; 95% CI: 3.48–12.64; *P* = 0.001).

The white areas of the PRCS test charts have a reflectance of 69.8%,^28,29^ and thus the luminance of these areas can be approximated to the illuminance multiplied by the reflectance divided by pi (that is mean luminances of 65.4 and 66.1 cd/m^2^; mean difference: 0.77; 95% CI: −0.48 to 2.02). The background luminance of the chart was therefore within a range for which the CS of the participant is known not to vary from that measured at the manufacturer's recommended background luminance of 85 cd/m^2^ (www.precision-vision.com/product/pelli-robsonsloanlettercontrastchart), owing to effects such as pupil miosis.^29^

The reflectance of the Sony Xperia Z series display has been measured to be approximately 5.6% (http://www.displaymate.com/Smartphone_ShootOut_3.htm). This implies that reflection of ambient light contributes approximately an added 5.4 cd/m^2^ to screen luminance, that is 5.6% of the illuminance divided by pi. The overall effect on brightness is therefore marginal.

The effect of such a reflection, if assumed to be uniform across the display, can be determined by re-calculating the log contrast sensitivities of each stage after adding 5.4 cd/m^2^ to the luminances measured in darkness. In each instance, this resulted in an increase in the CS measured by 0.009 log units. The effect of ambient light at the levels measured was therefore marginal; indeed PeekCS is less sensitive to fluctuations in ambient light than the Pelli-Robson chart and thus was disregarded in our subsequent analysis.

## Discussion

There are approximately 253 million persons with distance visual impairment worldwide.^30^ This number is based on standard VA measurement. It probably underestimates people who experience impaired visual function due to reduced CS.^3^ The current demographic trends of aging populations and increasing obesity will probably substantially increase in the global burden of visual impairment from macular degeneration, glaucoma, and diabetic retinopathy, conditions known to impair CS.^9–11,30^ Early detection of these conditions allows clinicians to intervene against sight loss.

CS is a sensitive measure of visual defects in glaucoma and is able to discriminate the severity of diabetic retinopathy and cataract.^10–12,31^ The traditional VA measure may not correlate well with day-to-day visual challenges that a person experiences or identify gradual neuropathological visual function changes.^5,32–34^ CS testing provides a measure of visual function that perhaps more readily reflects visual function in the “real world” and helps to identify more subtle or gradual vision loss.^3,5,33,34^ Some people, particularly at an older age, may have normal VA but reduced CS.^1,3^

Therefore, it is helpful to combine measurement CS with VA in clinical practice to obtain a more complete picture of visual function.^3^ However, CS is infrequently measured in routine clinical practice. This is particularly the case in low- and middle-income settings. The testing of CS requires training, equipment, and time. CS tends to be mostly used in research settings or specialist, very well-resourced clinics. Therefore, there is a need for a low-cost, accessible, quick, and easy to use CS test to enable more widespread use.

Increasing availability of smartphones, including in low and middle-income countries, provides a new opportunity to deliver CS testing. We have previously developed, tested, and released a smartphone-based application to test distance VA, Peek Acuity.^17^ This has been downloaded free of charge by more than 50,000 people in more than 140 countries. It is being used in teacher-delivered school-based screening programs to identify children in need of eye care services.^35^ Encouraged by this experience, we have developed the CS test described here.

PeekCS had very good test-retest reliability and was only slightly less well correlated than the test-retest of the PRCS test. The PeekCS measurements were highly correlated with the PRCS test and had 95% limits of agreement, which were equivalent to around two steps on the PRCS scale. The difference between the estimated mean CS scores between PeekCS and PRSC tests was very small and unlikely to be clinically significant as the upper bound of the 95% CI still only represents approximately one-seventh of a step in the PRCS test scale.

We believe that PeekCS offers a new CS test with several useful attributes. Most importantly, the test performance both in relation to the reference standard (PRCS) and test-retest is within clinically acceptable and useful limits. Compared with earlier mobile-device based CS tests, PeekCS is simple to use, validated in a relatively large sample with a wide range of CS.^18,19,21–25^ It is delivered on the relatively cheap Android-based smart phone platform. It uses the Tumbling-E design and therefore is more relevant to resource limited settings. The test time for PeekCS (45 seconds per eye) was somewhat shorter than those reported for other mobile application tests, which ranged between 53 seconds to several minutes.^18,19,22,24^ PeekCS presents only one randomly orientated “E.” This avoids crowding effects and the possibility of learning a sequence if the same chart is used more than once. It provides an easier testing scenario for both the observer and the tester than the PRCS, where multiple letters are presented to the participant in one chart.

Unlike the gratings-based CS tests, which have only two choices of target orientation, the PeekCS provides three-alternative forced choice target orientations, which will reduce guessing and improve repeatability.^24^ In the PRCS, the tester usually needs to point to the letter being tested, whereas in the PeekCS test the observer only needs to swipe in the direction indicated by the subject and does not need to see the letter being tested. This probably increases tester objectivity. PeekCS provides an automated CS score calculation. The smartphone's ambient light sensor alerts the tester to ambient light levels above a certain level. Although a smartphone may require occasional calibration checks, the PeekCS does not have some limitations of the PR chart such as fading print, difficulty in maintaining an even illumination, and reflections from the chart surface, which may influence the test results, making it a more practical tool for use in wider settings.^36^

This study has several limitations. The study was conducted in people affected by trachoma with average age of 50 years. This may limit the generalizability of the study results. In addition, a detailed ocular examination was not possible in this study population, as recruitment was conducted in a community clinic setting. Further validation studies across different populations and disease groups may be required. This study was performed using a single type of smartphone; additional work is needed to assess the test performance on other handsets. We used two separate PRCS charts with different random sequences of the letter “E” for test-retests for each eye. However, the participants were tested on each chart twice, which could lead to a potential learning effect. With regards to PeekCS, the use of a tripod stand places some limitations on how the test is deployed. The reliability of the test when deployed on mobile devices employing display technologies, which are less sensitive to viewing angle, such as organic light emitting diode displays, could be investigated. Similar to the PRCS, the PeekCS only measures approximation of the CS function (CSF) at one point (peak), which provides limited information about frequency-specific deficits.^19,25^

Overall, PeekCS is a repeatable, rapid, accessible, and easy to perform CS test that provides results that are highly comparable with the Pelli-Robson CS test. It provides a realistic approach for collecting CS testing data in the most basic of clinical settings, providing greater insight into an individual's visual experience. Moreover, it may open up new approaches to the early detection and monitoring of ocular disease.

## Supplementary Material

Supplement 1

Supplement 2

Supplement 3
