# Supplementary material for: Development and Validation of a Smartphone-based Contrast Sensitivity Test
Source: Transl Vis Sci Technol. 2019 Sep 13;8(5):13. doi: 10.1167/tvst.8.5.13 (PMC6743644; doi:10.1167/tvst.8.5.13)
Supplement: Supplement 1 [file tvst-08-04-34_s01.pdf]

Supplementary Material

Pelli-Robson Contrast Sensitivity Test

The Pelli-Robson Contrast Sensitivity **(**PRCS) chart presents letters of the same size but varying contrast. Pelli-Robson charts consist of capital letters of the same size, arranged horizontally in multiple rows. Each row has six letters, arranged in two sets of triplets. The letters of each triplet are of the same contrast. There are 16 triplets altogether. The contrast decreases with each successive triplet down the chart. The background is white.[1] The contrast sensitivity associated with each triplet is the reciprocal of the lowest contrast for which at least two letters in a group are correctly reported and is usually recorded as a logarithm with base 10 units. Thus,

$$Contrast\ Sensitivity = \log_{10}\left(\left|\frac{I_b}{I - I_b}\right|\right) \quad (1)$$

where I and I_b are the luminance of the letter and the background, respectively, and the contrast is defined as the Weber contrast. The contrast decreases in 0.15 log base 10 unit steps with each triplet. The first triplets begin with 0.00 log units and the last with a maximum possible contrast sensitivity of 2.25 log units. The standard PRCS chart is used at 1 metre, with each letter subtending at 2.8°.[1] In the PRCS test, the end point is the faintest triplet from which two of the three letters are identified correctly. In this study because of low literacy rates we used a 1 metre "Tumbling – E" PRCS chart (Precision Vision, Woodstock, IL, USA), in which capital "E" letters are presented in four random orientations: Down, Up, Left, and Right.

The PRCS was performed following the standard instruction provided with the chart. Two separate PRCS charts with different random sequences of the letter "E" in four orientations were used for test-retest for each eye. The charts were ordered from Precision Vision, Woodstock, IL, USA specifically for use in people with no literacy. The eyes were tested separately. The testing distance was 1 metre, with the center of the chart at the level of the participants' eyes. The test was conducted in a bright room with natural illumination, uniformly distributed across the chart, avoiding direct illumination with a lamp or sunlight, to prevent glare or reflection. The test was explained to the participant, who was instructed to make a single attempt to indicate the direction of each "E" letter with their hands. It was explained that on progressing down the chart the letters would be become more difficult to see, but that they should try to guess the direction of the "E" even when they find the letter is not visible. The participants were encouraged to continue until they failed to correctly indicate the direction of 2 / 3 letters in a triplet. The final CS score was given by the faintest triplet for which the directions of 2 / 3 letters were correctly identified, by referring to the scoring pad for each chart. The test duration was measured using a stopwatch, started from the time when the first letter was pointed to by the examiner to the end of the test for each eye being tested. The charts were handled carefully with clean hands and were carried to the field in their protective packaging.

Peek Contrast Sensitivity Test Development

The development of the smartphone-based PeekCS test went through multiple stepwise iterations. Within each development cycle we rapidly tested performance in a new group of study participants. As the PRCS test is probably the most widely used of those currently available, with a result and interpretation that is most familiar to clinicians, we aimed to develop a test with a compatible scalar output.[1] The test characteristics in the iterative development process were assessed in terms of the test-retest performance and prototype PeekCS performance relative to the PRCS test. The main results we present are those from the final optimized version. The following is a brief summary of the main test development observations that informed the choices that shaped the final version PeekCS.

During the PeekCS test development process we assessed two different testing distances, 70 cm and 1 metre. The presented optotype screen size was calibrated to subtend 2.8° for each testing distance. We found the PeekCS test-retest was better at 1 metre.

The PeekCS test randomly selects the orientation of the "E" as it proceeds. The observer records the subject's response by swiping the smartphone screen in the direction indicated by the hand movement of the subject. We examined a number of testing algorithms. As one of our subsidiary aims was to explore whether we could reliably simplify and shorten the test, relative to PRCS, we assessed the PeekCS test-retest and its performance relative to PRCS for a number of presentation/end-point options, including 1, 3 and 5 optotypes per CS level. We found that the test presenting 3 optotypes with an end point of 2 / 3 correct, comparable to the PRCS test, provided the best test-retest performance. As with the PRCS, participants were asked to guess the direction of the "E" even if they were not certain.

Display technologies are designed so that for a linear increase in the signal voltage across a picture element (pixel) the perceived brightness of that pixel also increases linearly. The perceived brightness of a stimulus can be modelled as being proportional to the cube root of a stimulus' luminance.[2] Therefore, display screens should ideally increase their brightness according to the inverse of this relationship in order to present a perceived linear increase in brightness and optimize the usage of bits in encoding displayed images.

The luminance of a pixel, I, is therefore found according to the following equation,

$$\boxed{I = Kv^{\gamma}} \quad (2)$$

where v is the input signal, K is a gain factor and γ is a display specific constant, commonly referred to simply as the display's "gamma". For the Android operating system, the 'ideal' gamma is specified as 2.2, K is typically 1.0 and v is one of 256 equally spaced values on a normalized scale. However, devices' displays will rarely, if ever, have a gamma of exactly 2.2 and will typically have a value slightly larger than this ideal value. To understand the true luminance of a display, or part thereof, based on the input signal alone it is therefore necessary to determine the display's gamma.

To determine the gamma for an optotype in the center of an otherwise white display, a black 15mm by 15mm square (the minimum area the photometer could resolve) was presented in the center of the display. The square was then lightened by increasing its red, green and blue (RGB) values by an equal amount using the Paint.net image editing software (dotPDN LLC, San Leandro, CA, USA). As these values were represented on a 256 point scale, it was assumed that an increase in these RGB values amounted to an equal increase in the corresponding display pixel's normalised input voltage (v in Equation 2). The display was also assumed to have a gain (K in Equation 2) of 1.0 which is typical in display applications. A sequence of these images was then combined into a single video using Microsoft Movie Maker (Microsoft Corp., Redmond, WA, USA), with each image presenting for seven seconds before progressing to the next in the sequence, and then playing this video using the phone's video player. Each image was designed so that the luminance calculated using Equation 2 was such that the contrast, relative to the background and calculated according to Equation 1, was 0.15 log units less than the preceding image. The presence any artefacts resulting from video compression were assessed by extracting frames from the video using Microsoft Movie Maker and conducting image analysis in the ImageJ image processing software package (National Institutes of Health, Bathesda, MD, USA). Specially, this involved visually inspecting the mean pixel intensity profile of a horizontal section of each frame selected for analysis each of which included part of the central square.

The luminance of the central square in the sequence was measured in a darkroom with a photometer (LS-100, Konica Minolta, Tokyo, Japan). The sequence was cycled three times for each of 10%, 30%, 50%, 70% and 100% display brightness settings.

Having assumed a gain of 1.0, the approximate gamma of the display was found using the Microsoft Excel Solver to run a Monte Carlo simulation to fit a gamma curve to the measured luminances. For the Samsung Galaxy SIII, gamma values for the different display brightness settings were found to be 2.2430, 2.2666, 2.1963, 2.3782 and 2.4257, respectively. For the Sony Xperia Z3, the gamma values were found to be 2.3258, 2.2956, 2.3205, 2.3048 and 2.3160, respectively. Given that none of these values fell outside of the range typical for display screens (1.8-2.5), 100% display brightness was chosen for the subsequent tests in order to reduce degradation of display contrast owing to the background illuminance encountered when deployed to the clinical setting in question. The gamma curves for each smartphone's display with 100% brightness setting are shown in Supplementary Figure S1.

The luminance measurements were then repeated but for a sequence of squares whose lightness were predicted to give the letter contrasts found on the PRCS chart. The measurements were then repeated with iterations of the square and background lightness until contrasts closest to that of the PRCS chart were found. In order to ensure consistency with the PRCS chart, only a fully white or slightly off-white background, that is where V_{in} > 245, was considered; as were pairings where the foreground shape was darker than the background. The final pairings of foreground and background greys are shown in Table 1. Owing to the finite number of foreground-background pairings available, an exact match with the log contrast sensitivities specified for the Pelli-Robson chart is not possible. In each case the difference between the two tests was less than one 0.15 log contrast step.

It is also important to note that the perceived luminance of a liquid crystal display (LCD), such as those in both smartphones in question, is highly sensitive to viewing angle.[3] Therefore, in order to standardize the test as much as possible the smartphone was mounted on a small tripod to fix the screen in a vertical plane, facing to the test subject. This substantially improved performance. We tested the PeekCS in a darkened room and in a more brightly lit room, and found that repeatability was slightly better in the latter.

Final Peek Contrast Sensitivity Test – Detailed Description

The final version of the PeekCS test, with results presented here, was performed using a Sony Xperia Z3 Compact smartphone with an Android 4.4 operating system. The smartphone was mounted on a tripod stand (Vivitar VIV-VPT-1252 Camera Tripod) after being fitted in a "Twist Grip" (Manfrotto TwistGrip Universal Phone Clamp). Eyes were tested separately. The testing distance was 1 metre. Screen brightness for the phone was set to 100%. One letter "E" was displayed at a time in 1 of 4 random orientations. The test started with maximal contrast.

The participant was asked to make a single attempt to point in the direction of the letter "E". The tester swiped the phone screen in the direction that the participant indicated, without looking at the phone screen. The participant was encouraged to guess the direction the letter was facing even when they thought the letter was not visible. A flowchart of the test logic is shown in Supplementary Figure S2. If the swipe matched the direction of the letter E then the CS was increased by one level according to the schedule in Table 1 and the orientation changed at random to one of the 3 other orientations. If the swipe did not match the direction of the letter E displayed then the CS level was maintained and the orientation changed at random to one of the 3 other orientations. This process was continued until either the highest CS level was reached or two incorrect swipes were recorded at the same level. In the case that the highest CS level was reached, the test was repeated at that level until either two correct swipes were recorded, at which point the test was stopped and a score of 15 returned, or else two incorrect swipes were recorded. If two incorrect swipes were recorded at any level then a letter E was then displayed at the previous CS level. Once two correct swipes were recorded at the same level over the entire test then this CS level was returned as being the participant's contrast sensitivity. At the end of the test, the application displays the log contrast sensitivity result, the average screen brightness level and the test time.

References

1. Pelli D, Robson J. The design of a new letter chart for measuring contrast sensitivity. *Clinical Vision Sciences* 1988;2:187-199.
2. Stevens SS. On the psychophysical law. *Psychological review* 1957;64:153.
3. Aslam TM, Murray IJ, Lai MYT, Linton E, Tahir HJ, Parry NRA. An assessment of a modern touch-screen tablet computer with reference to core physical characteristics necessary for clinical vision testing. *Journal of The Royal Society Interface* 2013;10.

Table S1 – Luminance of PeekCS test optotype and background for each score state, the Webber contrast between the two and the equivalent contrast sensitivity (CS) tested in log units. Luminance was measured in a dark-room using a LS-100 photometer (Konica-Minolta) pointed at a central 2.5x2.5mm square which alternated between the optotype and background grey RGB value.

Score Stage	Optotype Grey RGB value	Background Grey RGB value	Mean Optotype Luminance ± S.E. (cd/m^2)	Mean Background Luminance ± S.E. (cd/m^2)	Weber Contrast ± S.E.	Mean Log_{10}(CS) ± S.E.
1	0	255	6.964 ± 0.13	497.2 ± 0.37	0.9860±0.00003	0.006126±7x10^{-6}
2	164	255	177.1 ± 0.06	482.4 ± 0.23	0.6329±0.0006	0.1987±0.0004
3	197	254	260.6 ± 0.15	472.2 ± 0.10	0.4482±0.0004	0.3485±0.0004
4	216	255	328.3 ± 0.00	482.5 ± 0.09	0.3196±0.0002	0.4953±0.0003
5	229	255	373.8 ± 0.19	483.0 ± 0.24	0.2261±0.0006	0.6458±0.0012
6	237	255	404.8 ± 0.44	484.3 ± 0.35	0.1642±0.0012	0.7846±0.0031
7	242	254	414.4 ± 0.07	465.3 ± 0.23	0.1094±0.0005	0.9610±0.0021
8	246	255	440.3 ± 2.55	482.7 ± 2.87	0.0879±0.0080	1.056±0.039
9	248	255	462.2 ± 1.06	497.2 ± 0.37	0.0699±0.0008	1.155±0.003
10	250	254	449.4 ± 0.50	468.2 ± 0.12	0.0402±0.0011	1.396±0.012
11	250	253	415.4 ± 0.21	428.1 ± 0.50	0.0297±0.0013	1.527±0.018
12	252	255	486.5 ± 1.02	497.2 ± 0.37	0.0216±0.0007	1.666±0.010
13	246	248	412.3 ± 0.17	420.6 ± 0.00	0.0197±0.020	1.707±0.010
14	252	254	486.5 ± 1.02	492.1 ± 0.91	0.0115±0.0002	1.940±0.003
15	254	255	492.1 ± 0.91	497.2 ± 0.37	0.0102±0.0005	1.993±0.015
