# Supplementary material for: Development and Validation of a Smartphone-based Contrast Sensitivity Test
Source: Transl Vis Sci Technol. 2019 Sep 13;8(5):13. doi: 10.1167/tvst.8.5.13 (PMC6743644; doi:10.1167/tvst.8.5.13)

Figure S1: 'Gamma curves' describing the relationship between the signal input to a pixel(s), Vin, and its relative luminance for the displays of each smartphone. The 'ideal' relationship, that with a gamma of 2.2, specified for Android devices (green line) is shown for reference.

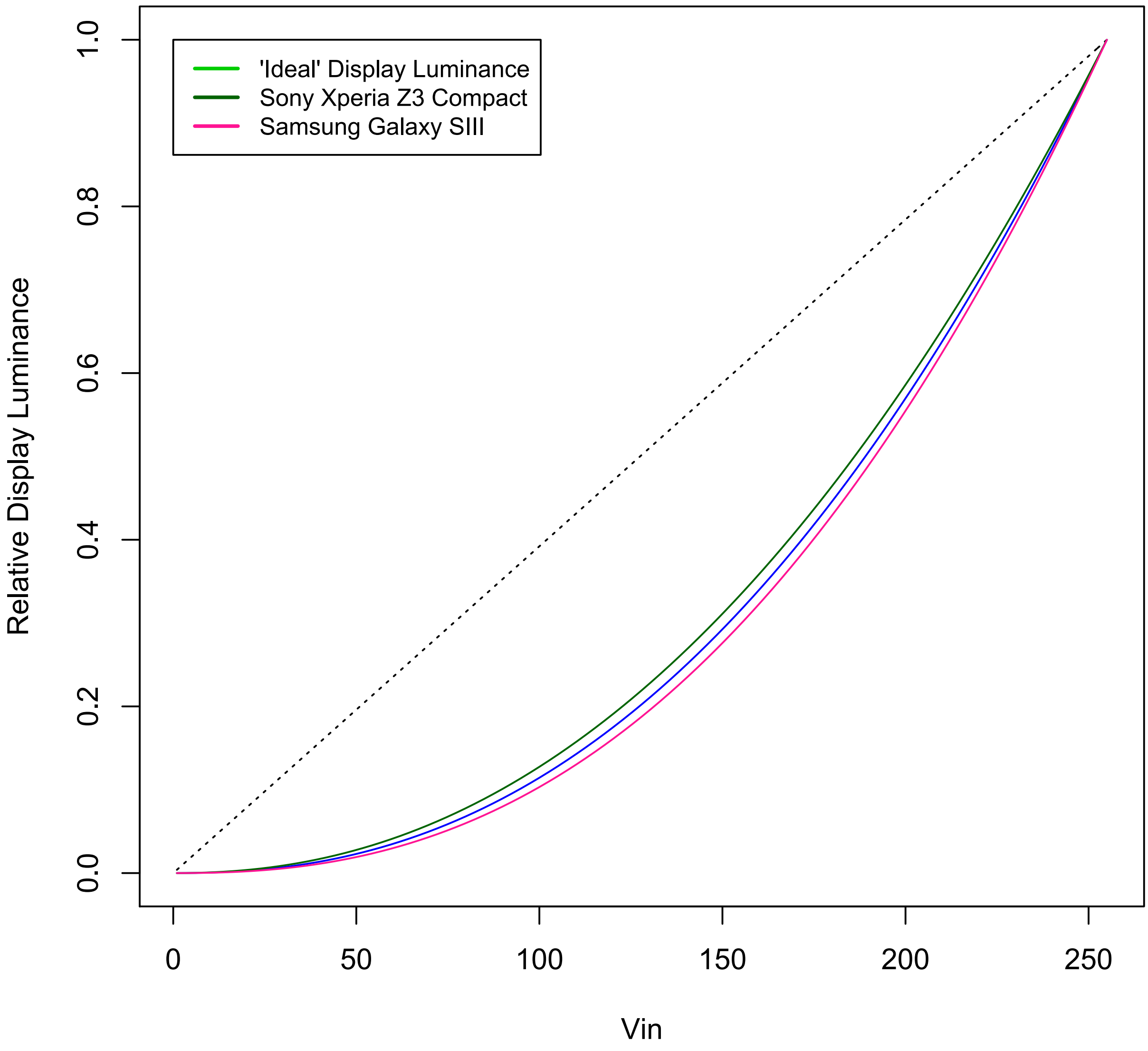

Supplement: Supplement 2 [file tvst-08-04-34_s02.pdf]
