# Supplementary material for: Development and Validation of a Smartphone-based Contrast Sensitivity Test
Source: Transl Vis Sci Technol. 2019 Sep 13;8(5):13. doi: 10.1167/tvst.8.5.13 (PMC6743644; doi:10.1167/tvst.8.5.13)

Figure S2: Flowchart describing the logic of the PeekCS test progression. Starting at stage 1, which has the highest contrast, the test presents tumbling Es testing for progressively higher contrast sensitivities (stages 2-15; the corresponding contrast sensitivities of which are shown in Table 1) upon the second instance where the direction of the E at a given contrast is correctly identified. In the event that the direction of two tumbling Es of the same stage cannot be identified correctly by the participant the test concludes, reporting a result equivalent to the base ten logarithm of the corresponding contrast sensitivity of the stage reached.

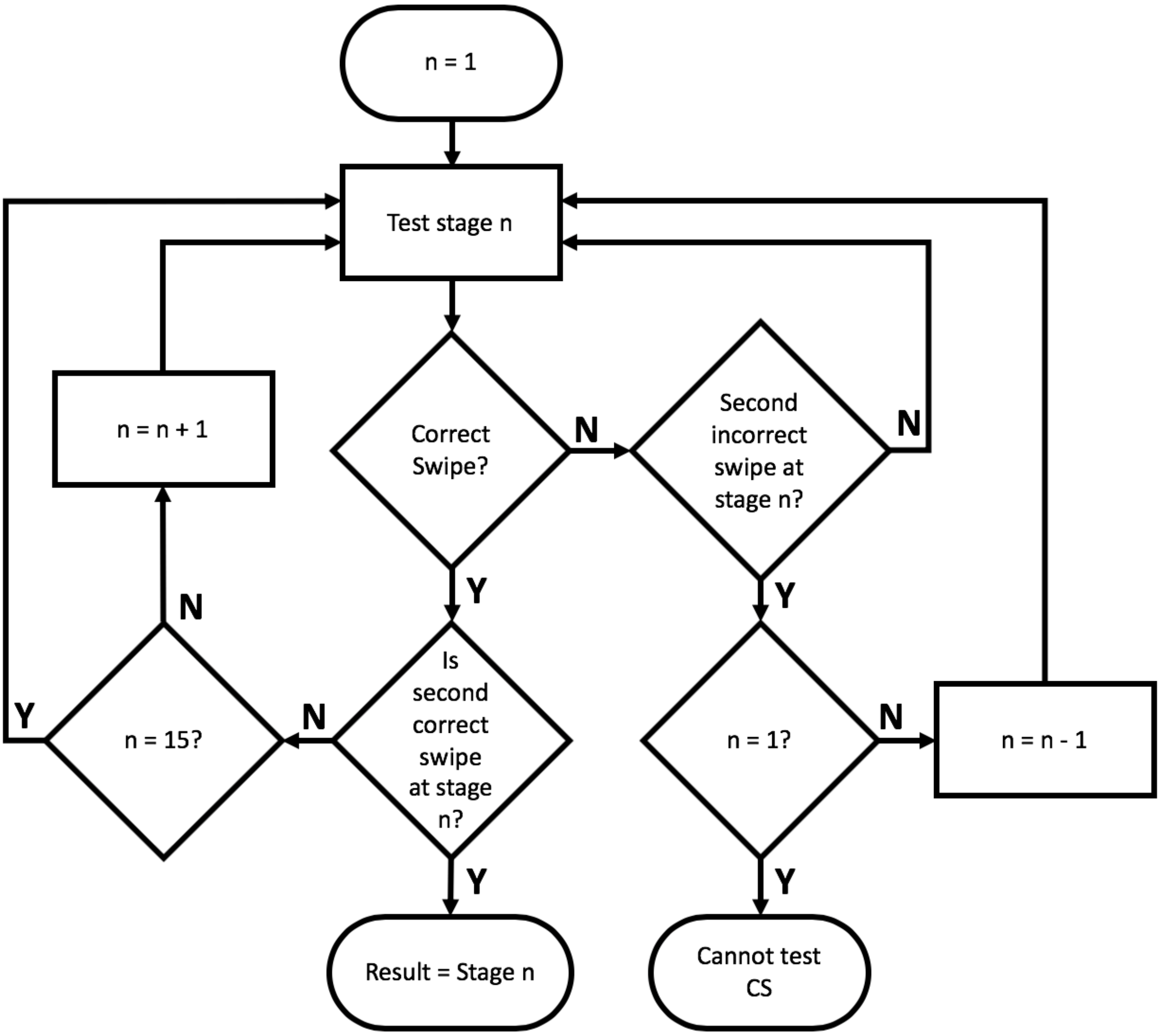

Supplement: Supplement 3 [file tvst-08-04-34_s03.pdf]
